# Cancer During Pregnancy: How to Handle the Bioethical Dilemmas?—A Scoping Review With Paradigmatic Cases-Based Analysis

**DOI:** 10.3389/fonc.2020.598508

**Published:** 2020-12-23

**Authors:** Diogo Alpuim Costa, José Guilherme Nobre, Susana Baptista de Almeida, Marisa Horta Ferreira, Inês Gonçalves, Sofia Braga, Diogo Pais

**Affiliations:** ^1^CUF Oncologia, Haematology and Oncology Department, Lisbon, Portugal; ^2^NOVA Medical School, Faculdade de Ciências Médicas, Lisbon, Portugal; ^3^Universidade de Lisboa, Faculdade de Medicina, Lisbon, Portugal; ^4^Hospital Professor Doutor Fernando Fonseca EPE, Oncology Department, Amadora, Portugal; ^5^Universidade da Beira Interior, Faculdade de Ciências da Saúde, Covilhã, Portugal; ^6^Hospital CUF Almada, Emergency Department, Almada, Portugal; ^7^Ethics Department, NOVA Medical School, Faculdade de Ciências Médicas, Lisbon, Portugal

**Keywords:** ethics, ethical, carcinoma, cancer, neoplasm, pregnancy, pregnant, gestation

## Abstract

Ethical issues that arise during the care of a pregnant woman with cancer are challenging to physicians, policymakers, lawyers, and the bioethics community. The main purpose of this scoping review is to summarize existing literature regarding the bioethical dilemmas when a conflict arises in the maternal-fetus dyad, like the one related to cancer and pregnancy outcomes. Moreover, we illustrate the decision-making process of real-life case reports. Published data were searched through the PubMed and Google Scholar databases, as well as in grey literature, using appropriate controlled keywords in English and Portuguese. After identification, screening, eligibility and data extraction from the articles, a total of 50 was selected. There are several established ethical frameworks for conflict resolution and decision-making. Pragmatic theoretical approaches include case-based analysis, the ethics of care, feminist theory, and traditional ethical principlism that scrutinizes the framework of autonomy, justice, beneficence, and non-maleficence. In addition, society and practitioner values could mediate this complex ethical interplay. The physician must balance autonomy and beneficence-based obligations to the pregnant woman with cancer, along with beneficence-based obligations to the fetus. Ethical challenges have received less attention in the literature, particularly before the third trimester of pregnancy. Best, unbiased and balanced information must be granted both to the patient and to the family, regarding the benefits and harms for the woman herself as well as for the fetal outcome. Based on a previously validated method for analyzing and working up clinical ethical problems, we suggest an adaptation of an algorithm for biomedical decision-making in cancer during pregnancy, including recommendations that can facilitate counseling and help reduce the suffering of the patient and her family.

## Background

Cancer is the second and first most common cause of death in women aged 25–34 and 35–65 years, respectively (1). However, cancer that occurs during pregnancy is a relatively rare event, with an estimate between 0.03 and 0.1% of all pregnancies. The incidence is expected to upsurge with later childbearing age and unplanned pregnancies. In Europe, 3,000–5,000 patients are diagnosed yearly with cancer during pregnancy, whereas 3,500 cases are reported in the USA (2–5). The most common neoplasms that occur during pregnancy are breast cancer, thyroid, cervical, ovarian and melanoma (2–5), but the currently available data is mainly limited to those areas of Western/Central Europe and North America (6, 7). Some recent data indicate that other cancers may be more prevalent in pregnant women in particular regions, as more cases of melanoma in Scandinavia (8) and gastrointestinal cancer in Asia (9).

The problematics of how to handle cancer during pregnancy has been a long-term matter of debate in the medical community. The many ethical issues that arise in the care of pregnant women involve many stakeholders—such as family, physicians, legislators, jurisdiction and the bioethics community - and its boundaries are imperfect since many contexts intersect. In the care of pregnant cancer women, it is important to consider the status of two biologically-related patients, but individually viable. However, cancer during pregnancy represents a dilemma given that treatment should be directed to keep two lives: maternal and fetal. Despite this complex ethical interplay, it should be emphasized, with the exception of special circumstances, that the patient has the final word in the decision-making process and that the remaining stakeholders contribute with a variable role and weight depending on each specific case and scenario.

This article focuses on the discussion to the clinical/pharmacological background and ethical issues that emerge from the medical management, especially before the third trimester, of a pregnant woman with cancer, which occurs whenever the therapy toxicity creates a conflict of interest that unbalances cancer and pregnancy outcomes. Furthermore, in order to permit a better framing of this problematic, we describe real-life paradigmatic cases that allow us to highlight the idiosyncrasies related to the ethical approach to cancer during pregnancy. Finally, we suggest an adaptation of an algorithm for biomedical decision-making in cancer during pregnancy, including some recommendations that can facilitate counseling and help reduce the suffering of the patient and her family.

## Methods

Assessing reasons or arguments presented in the normative bioethics’ literature can be a tricky task, as identifying any relevant data on a given topic in bioethics can be time-consuming and not always possible due to the high burden of grey literature, including books, edited book volumes and even predatory magazines, some of them with dubious content.

Therefore, some authors argue that, even if the systematic search should be maintained in bioethics research, the type of methodology will depend on the research question. In some cases, identifying all the literature on a given question may not be feasible and, even if it is, the time spent will not add significant value to the research (10, 11).

That is why some advocate a turn to critical interpretative reviews which might better serve bioethics research purposes (11). Based on that, we chose the scoping review as the best methodology for our research objectives, which were to rapidly map the existing literature (including the one not indexed in major databases, such as the grey literature), chart data from the studies, and clarify concepts. We were not interested in asking a single or precise question, but more focused on the identification of certain concepts in papers or studies, and in the mapping, reporting or discussion of the data collected. Our aim with this review is to summarize and clarify the existing published literature on the ethical dilemmas interwoven into the dimension of pregnant women with cancer, particularly before the third trimester. Moreover, we put in perspective the potential ethical problems and frameworks regarding the more appropriate approach for specific and representative case reports.

We developed an *a priori* protocol to define our research objective, and methods, which informed our selection for data extraction.

On October 8-9^th^, published literature was searched between 2010 and 2020 through the PubMed, using appropriate controlled keywords: [“ethics” (MeSH) OR “ethical” (MeSH)] AND [“carcinoma” (MeSH) OR “cancer” (MeSH) OR “neoplasm” (MeSH)] AND [“pregnancy” (MeSH) OR “pregnant” (MeSH) OR “gestation” (MeSH)]. References from the selected articles were scanned in order to identify other papers.

By the author’s decision, other relevant articles beyond this scope, including grey literature, have been included. For that purpose, we used Google Scholar search engine with the controlled words in English and Portuguese (“ethics” AND “cancer” AND “pregnancy”) OR (“ética” AND “cancro” AND “gravidez”), respectively.

Using Covidence (Covidence.org), we inputted our inclusion/exclusion criteria and selected the articles independently by two reviewers (DAC, JGN).

The inclusion criteria defined included: 1) patients who are pregnant; 2) patients who have active cancer; 3) articles addressing the problem of the triad: ethics, cancer and pregnancy; 4) articles including an ethical perspective during pregnancy; 5) articles and expert meeting reporting clinical practice guidelines or recommendations for cancer management during pregnancy; 6) study based on the toxicity of antineoplastic treatment during pregnancy that includes one of the following: chemo-, hormone-, targeted-, immuno-, and radio-therapy; 7) study based on the toxicity of supportive medication during pregnancy; 8) article language in English or Portuguese; 9) articles must be available with full-text.

The exclusion criteria were: 1) premenopausal women and fertility issues; 2) cancer risk exclusively after pregnancy; 3) cancer risk and hormone replacement therapy; 4) active cancer and breastfeeding; 5) articles discussing cancer therapeutics exclusively; 6) articles not mentioning pregnancy, cancer, and ethics at all.

Data extraction was conducted in Microsoft Excel version 16.41 (20091302) using a data charting form developed for our protocol.

## Results

Results were mostly restricted to review articles, ethical perspectives, clinical practice guidelines and case-based teaching guides (only available English and Portuguese text). Afterwards, the screening and selection of articles, quality assessment, and data extraction were performed independently by two reviewers (DAC, JGN), according to the pre-planned inclusion and exclusion criteria. Conflicts were resolved by a third party (IG) when a consensus was not reached.

**Figure 1:**
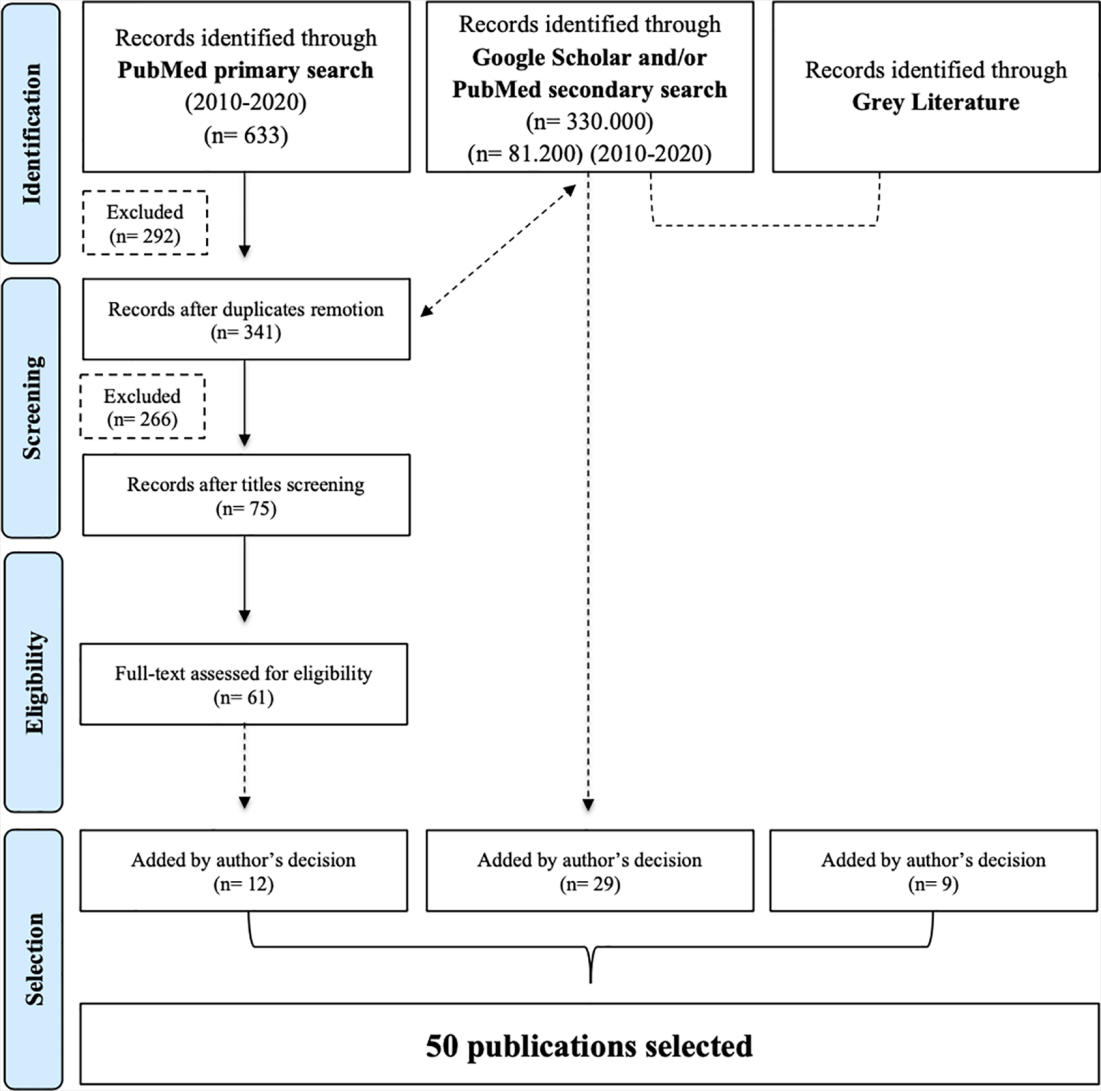
Flowchart explaining the article selection strategy (adapted from PRISMA, 2009).

For the period between 2010 and 2020, the initial search in English yielded 633 publications after the possible combinations of keywords in PubMed. Excluding the 292 duplicates, the number was reduced to 341. Title screen reduced the selection to 75 papers for reviewing abstracts and 61 fulfilled the criteria for reading the full-text. In the final selection, 12 articles were chosen to be included in this review (**Figure 1**).

The initial search in Google Scholar with the English keywords (“ethics” AND “cancer” AND “pregnancy”) resulted in 330.000 publications. By limiting the search between 2010 and 2020, 81.200 articles were retrieved. Furthermore, when searching for the Portuguese keywords (“ética” AND “cancro” AND “gravidez”), 3,150 papers were found. By limiting the search between 2010 and 2020, 2.680 publications were retrieved.

Twenty-nine additional articles in English and Portuguese, also indexed in PubMed, were included in this review and served as a reference to some of the previously searched articles. This second subgroup of articles included pioneering and relevant articles published in reference journals, as well as clinical trials and international guidelines/consensus.

Finally, a third subgroup of 10 publications was considered, which, despite not being indexed in databases, added value to the literature review. This group of articles was heterogeneous, with texts corresponding to international guidelines or to a health protection agency, book chapters or magazine sections (English and Portuguese), and case-based teaching guides.

## Discussion

### About Cancer During Pregnancy

The knowledge about the effect of cancer during pregnancy and the effect of cancer progression in pregnancy is of crucial importance for the success of the mother’s treatment and favorable outcome for the fetus. There are contradictory reports that have been published regarding the outcomes of these patients.

In 1880, Samuel Gross stated that breast cancer during pregnancy would behave like a rapidly growing disease, including with an “excessively malignant” clinical course (12). In 1943, after treating 20 patients with breast cancer, a group at Columbia University Presbyterian Hospital concluded that pregnancy “made the disease inoperable” (13). Ten years later, it was consensual that abortion was linked to improved patient survival (12). A population-based cohort study of 15,721 women diagnosed with breast cancer, of whom 1,110 (7%) had cancer during or within 2 years after pregnancy, revealed that this subset of patients had a worse prognosis, validating previous data (14). Conversely, a multicentric registry containing 447 women with breast cancer during pregnancy and 865 non-pregnant counterparts, showed similar overall-survival in both groups, after adjusting for known prognostic factors (15). Nonetheless, a careful interpretation of these studies should be taken, given the heterogeneity of the patient populations and treatments prescribed (16).

So far, there is no consolidated expert opinion on whether pregnancy can induce the occurrence or relapse of cancer and if it correlates only with maternal or also with other external or endogenous risk factors.

### Complementary Diagnostic Exams and Trimester Considerations

During the last decades, imaging of the pregnant patient has been performed with radiography, computed tomography, magnetic resonance imaging, scintigraphy, positron emission tomography scan, and ultrasonography (US). US imaging has emerged as the primary imaging modality because it provides real-time images without the use of ionising radiation (16).

A clear link between the severity of fetus impairment, gestational stage, and cumulative radiation dose received has already been established (16). For instance, during the organogenesis, there is a higher likelihood of major induction malformations and the threshold dose is above 100 mGy. There are also other issues besides ionising radiation. The radioactive iodine (I-131) crosses the placenta and has the ability to affect fetal thyroid and gadolinium teratogenic in animal studies. More invasive imaging tests should only be performed if the diagnosis and/or staging is expected to contribute decisively to the prognosis of the mother or fetus and that the risks and benefits are perfectly clarified and understood by the mother (16, 17).

Although the fetus is unscathed by laboratory tests, our main concern will be the influence that pregnancy will have on diagnosis, staging and follow-up, due to the fact that the serum biomarkers lack sensitivity and specificity during this period. There are tumor biomarkers that may be increased, such as CA 15-3, SCC, CA 125, and AFP, and others that are not so much, such as the example of CEA, CA 19-9, LDH, AMH, and HE-4. Inhibin B and LDH increased in the last trimester may be a laboratory sign of hypertensive abnormalities linked to pregnancy (4).

### Treatment Options and Trimester Considerations

The main challenge while managing cancer in pregnancy is balancing therapeutic regimen and fetus welfare. In addition, as an estimated 50% of pregnancies are unplanned, many women are exposed to teratogens before realising they are pregnant (13). This condition demands attention and careful protocols.

Approximately 0.5% of all births occur before the third trimester of pregnancy and the majority of these very early deliveries result in neonatal deaths and more than 40% in infant deaths. The delivery before 23 weeks of gestation, usually leads to neonatal death (5%–6% survival), and among rare survivors remains significant morbidity (98%–100%). When delivery is anticipated near the limit of viability, the patient, families and healthcare teams are faced with complex and ethically challenging decisions (18, 19). For most cytotoxic and targeted therapies, there is a lack of data regarding the risk of teratogenesis, based on case reports and retrospective series. The potential mutagenic, teratogenic and carcinogenic effects of ionising radiation and cytotoxic agents in the embryo are well known and depend on the dose, nature of the compound, treatment field and gestational stage. Some authors advocate that, if pregnancy occurs while the patient is under endocrine treatment (e.g., tamoxifen) or chemotherapy, a pregnancy termination should be recommended if it is done in the first trimester.

#### Surgery

In general, surgery can be performed during any stage of pregnancy with robust evidence demonstrating the safety of surgical procedures and most anaesthetic agents seem to be safe for the fetus. However, the risk of miscarriage is slightly incremented (1%–2%), especially in the first trimester. In addition, there is a higher risk of low birth weight and premature delivery (1.5–2 times relative risk), an increased rate of complications and higher morbidity in major abdominal and pelvic procedures. Relatively to anaesthetic drugs, there is a record of good safety and none of them stands in the drug list of proven teratogens. Given the fact that there is a minimal risk to the fetus and potential benefits of the treatment, there should not be any delay on the surgery, if indicated (2–4, 16).

#### Radiotherapy

The embryo-fetal risk can also be influenced by radiotherapy co-treatment and doses higher than 50–100 mGy should be avoided. Below these doses, there is a low risk of stochastic biological effects (mutations), and non-stochastic effects (malformations, developmental disorders) are as frequent as in general population (3%–5%) (2, 4, 20). In certain cases, it is necessary to use radiotherapy in the tumor, so the clinician must use it, in the period that it is least harmful to the fetus. From 2 to 12 weeks, the use of radiation has the risk of teratogenesis and growth retardation. Until 20 weeks, the fetus can present mental and growth retardation, microcephaly, eye, palate and genital deformities and beyond that, there is an increased risk of sterility, malignancies, and genetic defects (2–4, 16).

#### Chemotherapy

The most sensitive and critical period of drug exposure is organogenesis, which occurs roughly 2–8 weeks post-conception (17), especially during the gastrulation period when tissues are differentiating rapidly, and damage becomes vast and irreparable (21). Therefore, during the first trimester, the risk of spontaneous abortions, fetal death and major congenital malformations are increased, reaching 10%–20% and decline to about 6% when folate antagonists like methotrexate are excluded. The effects of antineoplastic agents during the second trimester are related to intrauterine growth restriction, low birth weight, miscarriage, and premature birth (20%–40%) (2–4). During the perinatal period, the effects are related to maternal/fetal myelosuppression, infections, and haemorrhage. Long-term outcomes of children exposed to chemotherapeutic agents in utero are not well examined. It is known that it is safe to give some drugs during the third trimester without causing long-term damage to the baby, for example, for Hodgkin´s disease or breast cancer (22).

#### Endocrine Treatment

In contrast to non-pregnant counterparts, pregnancy-associated breast cancer is more likely to develop higher stage tumors, more poorly differentiated, and less common oestrogen or progesterone-receptor positivity. These results were corroborated by previous studies. Nevertheless, there is still a significant fraction of hormone-receptors positive breast cancer (23).

However, many of the physiological changes during pregnancy are hormone-driven. Furthermore, the blockade of oestrogen (e.g., with tamoxifen), which is frequently used in hormone-positive breast cancer, might interfere with these physiological modifications and can be teratogenic and associated with fetal death and birth defects, mainly craniofacial anomalies (preauricular skin tags, microtia, hemifacial microsomia), ambiguous genitalia (clitoromegaly, labial fusion), and acetabular and sacral dysplasia. Tamoxifen is also associated with vaginal bleeding and miscarriage. Moreover, tamoxifen is not recommended during the lactation period, as it delays milk production, and there are limited safety data regarding its excretion in human milk. Importantly, the decision to postpone the tamoxifen to allow lactation should be based on individual risk and include a balanced discussion between risk and benefit (3, 4, 16, 20, 24). However, the tamoxifen effects on the fetus and the course of pregnancy are not yet fully understood (24).

#### Targeted Agents

Most of these targeted agents commonly used in breast cancer, such as trastuzumab, pertuzumab, bevacizumab, among others, should not be used because they present some undesirable adverse effects, but also due to the fact that there is missing much information yet. In general, human epidermal growth factor receptor 2 agents are safe during the first trimester, although during the second and third trimesters oligohydramnios, pre-term delivery and neonatal deaths may be present. Rituximab, an anti-CD20, imatinib, an anti-Bcr-Abl tyrosine kinase, and ATRA, a trans-retinoic acid, can be used with caution, even though they cross the placenta. Rituximab is safe in the first trimester, but in the coming trimesters, it causes cytopenia and B cell depletion, reversible at birth, while imatinib is safe in the second and third trimesters, with the risk of causing major malformations in the first trimester. ATRA is mainly dangerous in the first trimester due to the risk of abortion. The only targeted agent safe throughout pregnancy is interferon-α (3, 4, 16, 20, 25, 26).

#### Immunotherapy

A plethora of immunotherapy options is being used in the investigation and active treatment of several malignancies. As it is a more recent treatment, there is not much information regarding the security of these drugs during human pregnancy. However, as we all know, mother and fetus are not genetically identical. Therefore, an immunological tolerance from the mother towards the fetus is necessary in order for the pregnancy to develop successfully (26).

Immune checkpoints, such as programmed cell death protein-1 (PD-1), PD-1 ligand (PD-L1), and cytotoxic T-lymphocyte–associated protein 4, play a crucial role in the process aforenamed. Consequently, the fetus can be harmed by an aggressive immune response after the inhibition of these immune checkpoints. Furthermore, the drugs that can inhibit the checkpoints are immunoglobulins G4 antibodies that have the ability to cross the placenta and cause toxicity directly to the fetus. In animal models, these drugs demonstrated that their use could increase abortion rates, stillbirths, premature delivery and higher incidence of infant mortality, namely in the third trimester. However, there was not an increase in fetus malformations. In summary, since these drugs are so recent and have so little information regarding their security among pregnant women, immune checkpoint inhibitors are not recommended (26).

#### Supportive Medication

Our concern about pregnancy in women with cancer should not only focus on antineoplastic agents, but even on non-antineoplastic agents used in clinical cancer practice, such as bisphosphonates, granulocyte colony-stimulating factor (G-CSF) or granulocyte-macrophage colony-stimulating factor (GM-CSF), antiemetics, analgesics, and anti-inflammatories (2–4, 16, 20, 26).

Because bisphosphonates inhibit bone resorption, they are used in the treatment of hypercalcemia, osteoporosis, metastatic bone disease, and Paget disease. The bisphosphonates inhibit osteoclastic bone resorption *via* a mechanism that differs from that of other antiresorptive agents. In addition to their inhibitory effect on osteoclasts, bisphosphonates appear to have a beneficial effect on osteoblasts. These biological effects can lead to a reduction in serum calcium in the maternal blood and its availability to the fetus, which might induce skeletal malformations, reduced bone growth and low birth weight. It can, inclusively, affect parturition adversely by reducing uterine contractions. Therefore, it is contraindicated during pregnancy (3, 20).

G-CSFs/GM-CSFs are recommended in cases of severe neutropoenia or as primary/secondary prophylaxis during treatment with some chemotherapy regimens. Its safety during the pregnancy period is still unknown. In animal studies, it seems to cross the placenta and increase the rate of spontaneous abortion and low birth weight (2, 3). However, G-CSFs have already been used during pregnancy, without complications. Thus, these agents may also be considered during pregnancy if a high risk of neutropoenia is forwared (2, 3, 16, 26).

Antiemetics (metoclopramide, cyclizine, meclozine, alizapride, ondansetron, and aprepitant) can be safely used during the first trimester of pregnancy. The safety of corticosteroids is variable, with the use of hydrocortisone and prednisolone being preferred to dexamethasone or betamethasone, as they are extensively metabolized in the placenta and relatively little detected in the fetal compartment. Repeated administrations of betamethasone are associated with attention problems and cerebral palsy. Analgesics (paracetamol, opioids, anti-inflammatory agents), with the exception of the first trimester, did not generate side effects, but there is some risk of respiratory depression and fetal ductus arteriosus closure (3, 4, 16).

### The Ethical Issue: Balancing Interests

Pregnancy appears as an exceptional circumstance in medical ethics as the primary medical principle *Primum non nocere* can be questioned, as the access to the fetus occurs exclusively through intervention on the pregnant mother and treating the mother may imply harming the fetus. This is a unique situation since the welfare of both mother and fetus must be considered on any treatment planning.

When a conflict arises in the maternal-fetus dyad, caregivers must understand the pregnant woman´s mindset, broad social network, values, cultural, and religious beliefs, as this may impact their decisions (27). Consequently, it is imperative to promote the autonomy and physical integrity of the pregnant woman, ensuring that all available information on pregnancy and cancer outcomes is provided in order to allow for a fully informed consent consistent with her values (28) since the woman’s decision is absolute and unlimited. Therefore, in cases when the woman’s decision may harm her fetus (e.g., treatment of cancer during the first trimester) coercion to force treatment is never justified.

To date, there is no data systematically collected reporting the decision-making process of women who had cancer and pregnancy at the same time. Although, there are two studies conducted in the United Kingdom reporting patient experiences with participation in ORACLE (29) (evaluate the possibility that treatment with antibiotics prolongs labor and improves neonatal outcomes in women who are less than 37 weeks pregnant and experiencing either pre-term labor or premature rupture of membranes) and in the Magpie Trial (30) (prophylactic use of anticonvulsants for women with severe preeclampsia). In these studies, the major motivating factors were identified as self-benefit (it can help treat mother condition), benefit for your child (it can minimize the associated risks for the fetus) and altruism (participation can help future women or is it for the sake of medical science). It was also shown that, although some women seek the opinion of family and friends, they have little involvement or influence in women’s decisions. The partners played a role in providing a second opinion for many women, but study participants rejected the idea that their relatives or friends were in a position to influence their decision. In parallel, it may be reasonable to consider, in the future, the option of offering pregnant women with cancer the possibility of participating in clinical trials and/or enrolling in registries, increasing the motivation and the expectation of benefits for them and the fetus.

In the child-to-be perspective, there are extra layers of ethical complexity to address, because the antineoplastic treatment typically affects not only the pregnant woman but also the fetus. The developing fetus clearly has no capacity for autonomous choice, and there is no formula for balancing the interests and moral claims of the fetus with those of the mother. Furthermore, the welfare of the fetus is typically not independent of the interests of its mother (31).

When a conflict arises in the maternal-fetus dyad, such as cancer treatment and the risk of fetal demise, a range of ethical frameworks may be useful in the decision-making process. It is clear that the physician has beneficence-based and autonomy-based obligation to the pregnant cancer patient (32). Because of an immature central nervous system, the fetus cannot meaningfully be said to possess values and beliefs, although this is tremendously arguable.

Hence, scientifically there cannot be autonomy-based obligations to any fetus, although women’s beliefs may hasten her to judge differently (32). However, the physician can have beneficence-based obligations to the fetus, if the fetus is considered as a patient (33). The pregnant woman is free to withhold patient status, confer patient status, or, after conferring it, withdraw it from her pre-viable fetus (32). The fetus has no claim to patient status independently of the pregnant woman’s autonomy. When the woman is uncertain about or is not able to confer the status, the fetus can be provisionally regarded as a patient (32, 33). However, these approaches have been criticized for their tendency to emphasize the divergent rather than shared interests of the pregnant woman and the fetus. In fact, in most cases, the interests of the pregnant woman and fetus actually converge (28).

Whenever a pregnant woman is presented with a cancer diagnosis, several ethical concerns addressing technicalities must be approached, while keeping in mind the surrounding emotional issues.

There is no established *modus operandi* for the physician, which raises pertinent questions: i) should the patient be included in the decision-making to the best of her abilities in a limited way, or should paternalistic decision-making take over? ii) Should a proxy decision-maker decide based on the perceived patient’s best interest? (20) These are queries that can be carefully grasped by solving real-life cases, in parallel those that will be presented later.

To allow for an informed decision, the patient must be well aware of multiple medical facts, such as cancer prognosis, the possibilities of antineoplastic therapy, its main toxicities and its aim, namely: whether curative or palliative, if it will improve quality of life, or overall and progression-free-survival, if there is a risk of pre-term delivery or if peripartum complications are expected.

The timing of treatment must also be considered—is the mother symptomatic and needs to initiate treatment quickly or is it possible to delay it until the third trimester, when there is no significant risk for fetal defects in a short and long-term? Besides technical issues, before starting the treatment, the physician must consider emotional issues, such as the possibility of the child-to-be meeting its mother.

There are several established ethical frameworks for conflict resolution and decision-making. Pragmatic theoretical approaches include case-based analysis, the ethics of care, feminist theory, and traditional ethical principlism that scrutinizes the framework of autonomy, justice, beneficence, and non-maleficence. In addition, society and practitioner values could benefit this complex process.

### Illustrative Cases of Bioethical Dilemmas and a Proposed Algorithm for Ethical Decision-Making

For instance, the 1987 paradigmatic case of a pregnant woman in her late 20s, who had a late lung relapse 15 years after Ewing´s sarcoma diagnosis brought these issues to ahead. Although fully committed to saving her life, at the end of the second trimester, it became clear that the patient was dying. The Medical Centre tried to insist upon an early Cesarean section delivery in order to save her fetus. She refused the intervention with the support of her family, knowing it would almost certainly kill her, but the hospital forced the delivery through a court order. Both the patient and her extremely premature baby survived for only a short while after the surgery. In 1990, the Court of Appeals posthumously vacated the court-ordered Cesarean section, holding that the patient is totally autonomous to make healthcare decisions for herself and her fetus and that only in the most exceptional circumstances should a pregnant woman’s right to refuse interventions be called into question (34). Despite the media exposure of this case, others with similar ethical issues were far from being elucidated (35–37).

**Figure 2:**
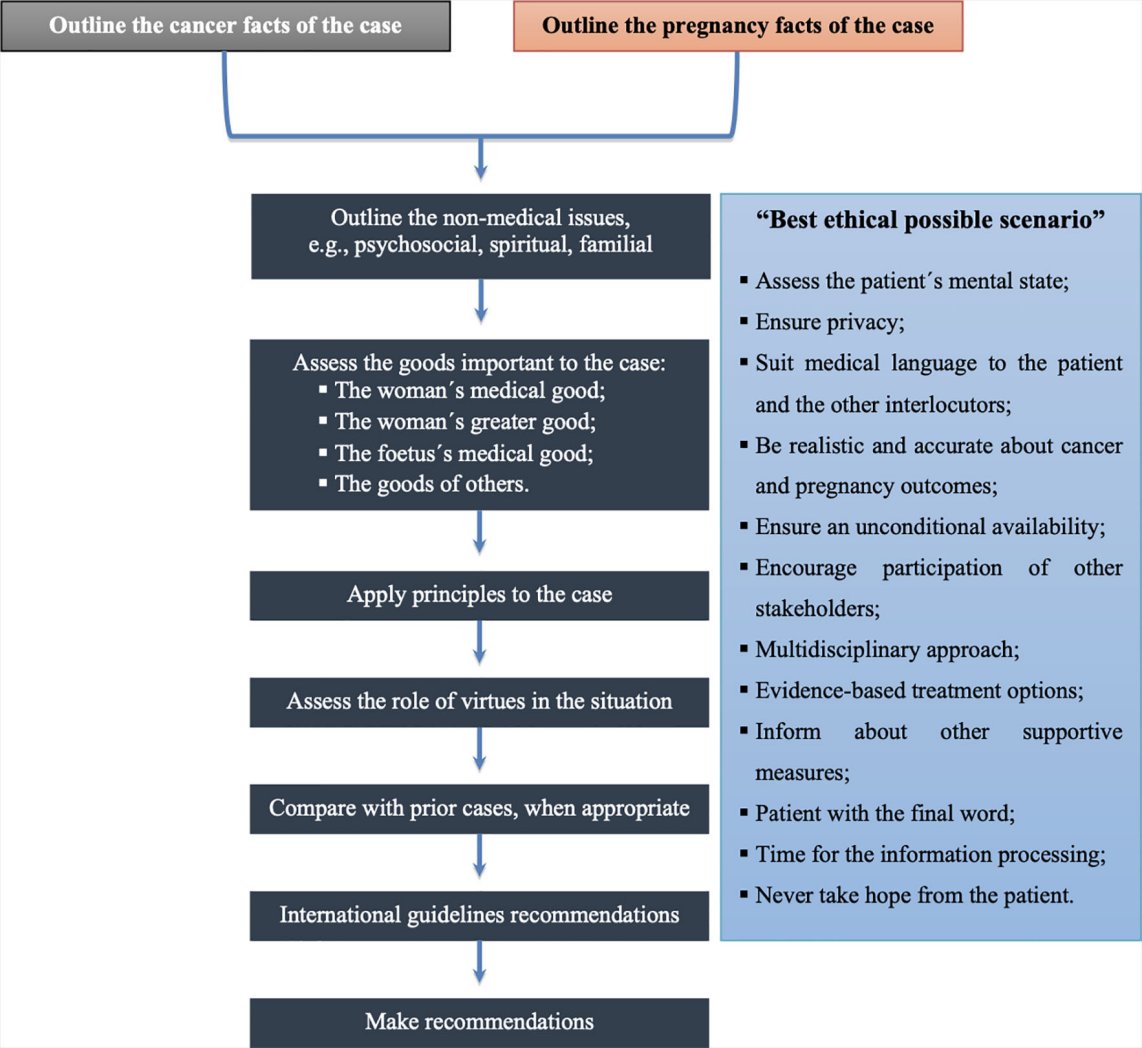
Algorithm for biomedical ethical decision-making (adapted from Schenck (38) and Botha et al. (39)).

This first case is an example of what should not be done in ethical terms when approaching such a complex and sensitive context. It was noticeable that there was no multidisciplinary management and strategy for a balanced approach to outline cancer and pregnancy facts of the case and the non-medical issues to achieve the best ethical possible scenario (**Figure 2**).

It is possible to infer that the woman would be in mental conditions to take a position. Hence, all the best possible information, at that moment, namely the cancer and pregnancy outcomes for the woman, evidence-based treatment options and available supportive measures for the situation, should have been presented to the patient. Furthermore, the comparison with previous similar ethical scenarios and the consultation of guidelines should have been assessed and discussed, in a multidisciplinary gathering, comprising the whole medical specialities related to this subject (e.g., Medical Oncology, Obstetrics, among others), a representative of the ethics committee, all the stakeholders (such as the patient’s family and the fetus’ father) and the patient itself. Finally, the patient’s final decision, in collusion with the autonomy ethic’s principle, should have been respected.

In 2016, on February 20, a 17-week-pregnant woman in her late 30s, collapsed after an intracerebral haemorrhage, probably related to a late recurrence of kidney cancer diagnosed 10 years earlier (40). Soon after physicians declared her brain dead (41). The hospital ethics committee and the family were enquired. It was explained to both parties - mother’s family and the fetus’ father—that, to allow for the fetus survival, the woman should be kept on life-sustaining treatment to reach at least its 32 weeks, the earliest date doctors felt that a successful Cesarean delivery would be possible (40). This emphasizes the role of the “mother’s body as a cadaveric incubator”, “mother as the organ donor and fetus as the recipient”, and the concern for “possible damages to the fetus” (42–44). Some professionals believe that it is not ethically acceptable to maintain the mother’s body after brain dead to use it as a “fetal container.” Such a decision should not be assumed, but it must be debated. If the mother is to be considered a “cadaveric incubator” with no autonomous rights, the rights of the fetus should legally prevail. Another argument claims that the continued somatic support itself is actually organ donation with the fetus as the recipient (42–44). The family strongly expressed that the mother would have wanted her life preserved in order to give the fetus a chance for survival. The ethics committee equated the fetus life to a child at risk and allowed the support to the brain-dead mother. The decision was taken in a meeting of the neurosurgical, critical care, obstetrics, neonatal, transplant and ethical staff, along with the patient’s family. One hundred seven days later, the baby was born healthy, and the life-sustaining machines were turned off (40, 41).

About 10 years earlier, a woman of the same age and gestational stage as in the previous case suffered a stroke secondary to the brain progression of melanoma. Soon after, the doctors declared brain dead. Her family also decided to keep her alive to give the fetus a chance. It became a race between the fetus’ development and cancer that was ravaging the patient’s body. The baby was born about 2 months prematurely, and tragically did not survive. At that time, the case was also considered to be notable because there was no controversy (43).

Put into perspective, the two aforementioned cases have analogous ethical issues, however, with different endings. Nevertheless, both these situations follow the best ethical possible scenario approach (**Figure 2**). Since both patients were declared brain dead, none of them would be mentally suitable to take a position. Thus, the patient’s family and the fetus’ father should be responsible for the decisions regarding equally the patient and the fetus welfare. As observed, in these circumstances, all of the stakeholders had received the best information possible *vis-à-vis* the cancer and pregnancy outcomes, the evidence-based treatment options to follow pregnancy and supportive measures for maintenance of “cadaveric incubator”, with the aim for the survival of the fetus. All the decisions were engaged with a multidisciplinary team, taking into consideration previous comparable clinical cases, and respecting the patient’s family and fetus’ father autonomy. Since the number of cases describing the management of extended maternal somatic support after brain death is limited, every case should be continuously reassessed and adapted along with the increasing experience and knowledge (42–44).

In these difficult cases, mainly before the third trimester, the sovereign decision should be taken after thorough discussion between mother (or legal substitute) and the treating physician (42–46). While respecting the principle of autonomy, another final ethical issue is the right of the physicians to conscientiously object to certain treatment options.

As stated before, physicians should not bias with their recommendations and should present to consider three scenarios: i) treatment during pregnancy, with close monitoring for side effects and reconsideration of termination before viability; ii) treatment with termination of the pregnancy; and iii) treatment delay until fetal lung maturity, when it’s reasonably safe to deliver the baby (34, 47).

All over Europe, there are differences between countries regarding availability, conditions and gestational limit. In Portugal, since 2007, and after a National Referendum, the voluntary termination of pregnancy until 10 weeks of gestation was legalized (law n°16/2007). In that same legal document, it is stated that in case of danger of death or physical and/or psychic injury, the possibility of interrupting the pregnancy until 12 weeks of gestation is allowed (48).

The available international guidelines recommend that maternal fetal medicine consultation should include counseling on maintaining or terminating a pregnancy, including a review of the treatment options. These guidelines support a framework of shared decision-making in the context of maternal-fetal conflict to provide guidance for compassionate conflict resolution. An ethics consultation may be helpful to mediate conflict resolution. Intervention by the courts is rarely appropriate or indicated and should be avoided (27, 47, 49).

Based on a validated method for analysing and working up clinical ethical problems, we suggest an adaptation of an algorithm for biomedical decision-making in pregnancy-associated cancer (**Figure 2**).

The first task in this ethical decision-making process is to establish the medical and pregnancy facts of the case. The second step is to determine pertinent non-medical issues, which is more challenging. These steps are followed by an assessment of the goods relevant in the case. The immediate concern is clearly what is suitable for the woman medically, but that is followed closely by an attempt to understand the patient’s overall good—e.g., psychological good, good in terms of family and relations, spiritual good, and good in terms of the patient’s preceding life history and values. While ensuring the good of the woman is the primary aim, this is insufficient in itself, as the goods of fetus and others must also be considered (38).

The principles that apply in the case at hand are then evaluated, specifying what a given principle means in this case and balancing it against the moral claims of each of the others. In themselves, principles can become mere abstractions, perhaps even sterile nostrums for dealing with these complex ethical dilemmas. Therefore, virtue ethics, another bioethical approach that has received increased attention in recent years, addresses the nature of the relationship between patient and healer, with particular attention to the character of the physician (38). Pellegrino and Thomasma have presented a detailed analysis of how they interpret the virtues that are essential to medical practice. These virtues include phronesis, compassion, fidelity, trust, integrity, self-effacement, justice, fortitude, and temperance (50).

In any case, a consideration of the virtues and principles on the one hand, and guidelines recommendations and prior similar cases analysis on the other, provide more guidance for a right answer to bioethical dilemmas.

Finally, the guidelines recommendations should be accompanied by several steps that must take place in the “best ethical possible scenario”, in order to enhance the quality of the counseling and emotional support that are an essential part of management (**Figure 2**):

- Assess the mental state of the patient;
- Ensure privacy;
- Suit medical language to the patient and the other interlocutors;
- Be realistic and accurate about cancer and pregnancy outcomes;
- Ensure an unconditional availability for the discussion and re-discussion of each dough or clarification.
- Encourage participation in the decision-making process of the partner and the closest family members;
- Inform that medical management is not the responsibility of a single professional, but of a multidisciplinary team with a holistic approach;
- Provide evidence-based treatment options;
- Inform about other supportive measures;
- Inform that the patient has the final word in the decision-making process;
- Provide the necessary time for the information processing phases according to Kübler-Ross model (denial, anger, bargaining, depression, acceptance);
- Never take hope from the patient.

## Conclusions

Scientific and clinical data addressing the risks and the efficacy of treating a pregnant woman with cancer have already been explored in the literature. However, the imbricated ethical challenges have received less attention, particularly before the third trimester of pregnancy.

A pregnant woman with cancer faces the choice between best antineoplastic treatment versus maximal fetal welfare. Best, unbiased and balanced information about the benefits and harms for the woman herself as well as for fetal outcome must be granted both to the patient and to the family.

Halting, in this scoping review, the authors identified certain concepts and discussed the heterogeneous data collected regarding bioethical decisions on cancer during pregnancy. Nevertheless, there are still some unsolved queries, that must be discussed in multidisciplinary groups, and personalized to each unique scenario. Each new decision should be included in an updated shared and anonymous database to put in perspective what should be done in a particular cancer situation that affects two unique lives (maternal and fetal), allowing to gather attitudes and experiences that fill a knowledge gap needed to develop ethical care guidelines.

## Author Contributions

All authors have read the submission and agreed to submit this manuscript. The present manuscript is the result of original work by the co-authors. Conception and design: DAC, JN. Development of methodology: DAC, JN. Acquisition, analysis, and interpretation of data: DAC, JN, IG. Writing, review, and/or revision of the manuscript: DAC, JN, SD, MF, IG. Manuscript supervision: SB, DP.

## Conflict of Interest

The authors declare that the research was conducted in the absence of any commercial or financial relationships that could be construed as a potential conflict of interest.

## Abbreviations

AFP, Alpha-fetoprotein; AMH, Anti-Müllerian hormone; ATRA, All-trans-retinoic acid; Bcr-Abl, Breakpoint cluster region protein-Abelson murine leukaemia viral oncogene homolog 1; CA, Carbohydrate antigen; G-CSF, Granulocyte colony-stimulating factor; GM-CSF, Granulocyte colony-stimulation factor; HE-4, Human epididymis protein-4; I-131, Radioactive iodine; LDH, Lactate dehydrogenase; mGy, mili Gray units; PD-1, Programmed cell death protein-1; PD-L1, Programmed cell death protein-ligand 1; SCC, Squamous cell carcinoma; US, Ultrasonography; USA, United States of America.

## References

[1] Leading causes of death by age group, all females-United States 2013, The Center for Disease Control (CDC). 10.3978/j.issn.2072-1439.2015.AB015 Available at: http://www.cdc.gov/women/lcod/2013/womenall_2013.pdf (Accessed October 6, 2020).

[2] Azim H Jr Gentilini O Locatelli M Ciriello E Scarfone G Peccatori F Managing pregnant women with cancer: personal considerations and a review of the literature. Ecancermedicalscience (2011) 5:204. 10.3332/ecancer.2011.204 22276051 PMC3223950

[3] Maggen C van Gerwen M Van Calsteren K Vandenbroucke T Amant F Management of cancer during pregnancy and current evidence of obstetric, neonatal and pediatric outcome: a review article. Int J Gynecol Cancer (2019) 18:ijgc–2018-000061. 10.1136/ijgc-2018-000061 30659032

[4] Zubor P Kubatka P Kapustova I Miloseva L Dankova Z Gondova A Current approaches in the clinical management of pregnancy-associated breast cancer-pros and cons. EPMA J (2018) 9(3):257–70. 10.1007/s13167-018-0139-5 PMC6107452 30174762

[5] Mitrou S Zarkavelis G Fotopoulos G Petrakis D Pavlidis N A mini review on pregnant mothers with cancer: A paradoxical coexistence. J Adv Res (2016) 7(4):559–63. 10.1016/j.jare.2016.01.004 PMC4921772 27408757

[6] Loibl S Han SN von Minckwitz G Bontenbal M Ring A Giermek J Treatment of breast cancer during pregnancy: an observational study. Lancet Oncol (2012) 13(9):887–96. 10.1016/S1470-2045(12)70261-9 22902483

[7] Córdoba O Llurba E Saura C Rubio I Ferrer Q Cortés J Multidisciplinary approach to breast cancer diagnosed during pregnancy: maternal and neonatal outcomes. Breast (2013) 22(4):515–9. 10.1016/j.breast.2012.10.005 23116970

[8] Andersson TM Johansson AL Fredriksson I Lambe M Cancer during pregnancy and the postpartum period: A population-based study. Cancer (2015) 121(12):2072–7. 10.1002/cncr.29325 25737403

[9] Shim MH Mok CW Chang KH Sung JH Choi SJ Oh SY Clinical characteristics and outcome of cancer diagnosed during pregnancy. Obstet Gynecol Sci (2016) 59(1):1–8. 10.5468/ogs.2016.59.1.1 26866029 PMC4742470

[10] McDougall R Systematic reviews in bioethics: types, challenges, and value. J Med Philos (2014) 39(1):89–97. 10.1093/jmp/jht059 24334289

[11] McDougall R Reviewing Literature in Bioethics Research: Increasing Rigour in Non-Systematic Reviews. Bioethics (2015) 29(7):523–8. 10.1111/bioe.12149 25655982

[12] Pamela P With Child, With Cancer (2008). The New York Times Magazine Available at: https://www.nytimes.com/2008/08/31/magazine/31cancer-t.html?referer.%2520Accessed%2520September%252014,%25202019 (Accessed October 6, 2020).

[13] Haagensen CD Stout AP Carcinoma of the breast: ii. Criteria of operability. Ann Surg (1943) 118(5):859–70. 10.1097/00000658-194311850-00008 PMC1617728 17858315

[14] Johansson AL Andersson TM Hsieh CC Cnattingius S Lambe M Increased mortality in women with breast cancer detected during pregnancy and different periods postpartum. Cancer Epidemiol Biomarkers Prev (2011) 20(9):1865–72. 10.1158/1055-9965.EPI-11-0515 21750168

[15] Amant F von Minckwitz G Han SN Bontenbal M Ring AE Giermek J Prognosis of women with primary breast cancer diagnosed during pregnancy: results from an international collaborative study. J Clin Oncol (2013) 31(20):2532–9. 10.1200/JCO.2012.45.6335 23610117

[16] Shachar SS Gallagher K McGuire K Zagar TM Faso A Muss HB Multidisciplinary Management of Breast Cancer During Pregnancy. Oncologist (2017) 22(3):324–34. 10.1634/theoncologist.2016-0208 PMC5344634 28232597

[17] Koren G Pastuszak A Ito S Drugs in pregnancy. N Engl J Med (1998) 338(16):1128–37. 10.1056/NEJM199804163381607 9545362

[18] Ferrari F Faccio F Peccatori F Pravettoni G Psychological issues and construction of the mother-child relationship in women with cancer during pregnancy: a perspective on current and future directions. BMC Psychol (2018) 6(1):10. 10.1186/s40359-018-0224-5 29548301 PMC5857118

[19] Obstetric Care Consensus No. 4: Periviable Birth. Obstet Gynecol (2016) 127(6):e157–69. 10.1097/AOG.0000000000001483 27214190

[20] Del Pup L Peccatori FA Azim HA Jr Michieli M Moioli M Giorda G Obstetrical, fetal and postnatal effects of gestational antiblastic chemotherapy: how to counsel cancer patients. Int J Immunopathol Pharmacol (2012) 25(2 Suppl):33S–46S. 10.1177/03946320120250s203 23092518

[21] Esposito S Tenconi R Preti V Chemotherapy against cancer during pregnancy: A systematic review on neonatal outcomes. Med (Baltimore) (2016) 95(38):e4899. 10.1097/MD.0000000000004899 PMC5044906 27661036

[22] Eyre TA Lau IJ Mackillop L Collins GP Management and controversies of classical Hodgkin lymphoma in pregnancy. Br J Haematol (2015) 169(5):613–30. 10.1111/bjh.13327 25684034

[23] Dotters-Katz S McNeil M Limmer J Kuller J Cancer and pregnancy: the clinician’s perspective. Obstet Gynecol Surv (2014) 69(5):277–86. 10.1097/OGX.0000000000000068 25101693

[24] Buonomo B Brunello A Noli S Miglietta L Del Mastro L Lambertini M Tamoxifen Exposure during Pregnancy: A Systematic Review and Three More Cases. Breast Care (Basel) (2020) 15(2):148–56. 10.1159/000501473 PMC7204783 32398983

[25] Lambertini M Peccatori FA Azim HA Jr Targeted agents for cancer treatment during pregnancy. Cancer Treat Rev (2015) 41(4):301–9. 10.1016/j.ctrv.2015.03.001 25795021

[26] Azim HA Jr Pavlidis N Peccatori FA Treatment of the pregnant mother with cancer: a systematic review on the use of cytotoxic, endocrine, targeted agents and immunotherapy during pregnancy. Part II: Hematological tumors. Cancer Treat Rev (2010) 36(2):110–21. 10.1016/j.ctrv.2009.11.004 20018452

[27] ACOG Committee on Ethics ACOG Committee Opinion #321: Maternal decision making, ethics, and the law. Obstet Gynecol (2005) 106:(5 Pt 1):1127–37. 10.1097/00006250-200511000-00058 16260545

[28] Townsend S Maternal-Fetal Conflict. American Academy of Pediatrics Bioethics Resident Curriculum: Case-Based Teaching Guides(2017). Available at: https://www.aap.org/en-us/Documents/Bioethics-FullCurriculum.pdf (Accessed October 6, 2020).

[29] Kenyon S Dixon-Woods M Jackson CJ Windridge K Pitchforth E Participating in a trial in a critical situation: a qualitative study in pregnancy. Qual Saf Health Care (2006) 15(2):98–101. 10.1136/qshc.2005.015636 16585108 PMC2464828

[30] Smyth RM Jacoby A Elbourne D Deciding to join a perinatal randomised controlled trial: experiences and views of pregnant women enroled in the Magpie Trial. Midwifery (2012) 28(4):E478–85. 10.1016/j.midw.2011.08.006 21944570

[31] Coverdale JH McCullough LB Chervenak FA Assisted and surrogate decision making for pregnant patients who have schizophrenia. Schizophr Bull (2004) 30(3):659–64. 10.1093/oxfordjournals.schbul.a007113 15631258

[32] McCullough LB Chervenak FA Ethics in Obstetrics and Gynecology. New York: Oxford University Press (1994).

[33] Fancher KM Giannetti V McLaughlin BT Ethics of chemotherapy during pregnancy. Am J Health Syst Pharm (2019) 76(4):242–6. 10.1093/ajhp/zxy041 30715184

[34] Chervenak FA McCullough LB Knapp RC Caputo TA Barber HR A clinically comprehensive ethical framework for offering and recommending cancer treatment before and during pregnancy. Cancer (2004) 100(2):215–22. 10.1002/cncr.11564 14716752

[35] Kukla R Wayne K Pregnancy, Birth, and Medicine. The Stanford Encyclopedia of Philosophy . Available at: https://plato.stanford.edu/entries/ethics-pregnancy/ (Accessed October 6, 2020).

[36] Thornton TE Paltrow L The rights of pregnant patients: Carder case brings bold policy initiatives. Healthspan (1991) 8(5):10–6. 10111987

[37] Draper H Women, forced caesareans and antenatal responsibilities. J Med Ethics (1996) 22(6):327–33. 10.1136/jme.22.6.327 PMC1377113 8961116

[38] Schenck DP Ethical considerations in the treatment of head and neck cancer. Cancer Control (2002) 9(5):410–9. 10.1177/107327480200900506 12410180

[39] Botha MH Rajaram S Karunaratne K Cancer in pregnancy. Int J Gynaecol Obstet (2018) 143(Suppl 2):137–42. 10.1002/ijgo.12621 30306590

[40] Martins C José BebédoS seis razões para um milagre meritório (2016). Expresso Available at: http://expresso.sapo.pt/sociedade/2016-06-09-Bebe-do-Sao-Jose-seis-razoes-para-um-milagre (Accessed October 6, 2020).

[41] Correia A Manter os órgãos em funcionamento de uma mãe em morte cerebral é um objetivo meritório (2016). Visão Available at: http://visao.sapo.pt/actualidade/sociedade/2016-06-08-Manter-os-orgaos-em-funcionamento-de-uma-mae-em-morte-cerebral-e-um-objetivo-meritorio (Accessed October 6, 2020).

[42] Esmaeilzadeh M Dictus C Kayvanpour E Sedaghat-Hamedani F Eichbaum M Hofer S One life ends, another begins: Management of a brain-dead pregnant mother-A systematic review-. BMC Med (2010) 8:74. 10.1186/1741-7015-8-74 21087498 PMC3002294

[43] Gregorian A Post-mortem pregnancy: a proposed methodology for the resolution of conflicts over whether a brain dead pregnant woman should be maintained on life-sustaining treatment. Ann Health Law (2010) 19(2):401–24, preceding i. 21443149

[44] Sheikh AA Cusack DA Maternal brain death, pregnancy and the foetus: the medico-legal implications for Ireland. Med Law (2004) 23(2):237–50. 15270467

[45] Manos E Gkika D Euthimiou C Lola V Potonos S Kokkori I Ethical dilemmas, medical protocols and deontology in diagnosis of lung cancer during pregnancy. J Thorac Dis (2015) 7(S1):AB015. 10.3978/j.issn.2072-1439.2015.AB015PMCID:PMC4332062

[46] Policicchio D Doda A Muggianu G Dipellegrini G Boccaletti R Ethical and therapeutic dilemmas in glioblastoma management during pregnancy: Two case reports and review of the literature. Surg Neurol Int (2019) 10:41. 10.25259/SNI-86-2019 31528379 PMC6743686

[47] National Comprehensive Cancer Network (NCCN) Practice Guidelines in Oncology(2020). Available at: https://www.nccn.org/professionals/physician_gls/pdf/breast.pdf (Accessed October 10, 2020).

[48] Vicente LF The woman’s choice for abortion: the experience in Portugal with implementation of the National Network. Cad Saude Publi (2020) 36Suppl 1(Suppl 1):e00036219. 10.1590/0102-311X00036219 32267398

[49] Peccatori FA Azim HA Jr Orecchia R Hoekstra HJ Pavlidis N Kesic V ESMO Guidelines Working Group. Cancer, pregnancy and fertility: ESMO Clinical Practice Guidelines for diagnosis, treatment and follow-up. Ann Oncol (2013) 24(Suppl 6):vi160–70. 10.1093/annonc/mdt199 23813932

[50] Pellegrino ED Thomasma DC The Virtues in Medical Practice. New York, NY: Oxford University Press (1993).

